# Integrating High-Throughput Phenotyping and Statistical Genomic Methods to Genetically Improve Longitudinal Traits in Crops

**DOI:** 10.3389/fpls.2020.00681

**Published:** 2020-05-26

**Authors:** Fabiana F. Moreira, Hinayah R. Oliveira, Jeffrey J. Volenec, Katy M. Rainey, Luiz F. Brito

**Affiliations:** ^1^Department of Agronomy, Purdue University, West Lafayette, IN, United States; ^2^Department of Animal Sciences, Purdue University, West Lafayette, IN, United States

**Keywords:** digital agriculture, genomic estimated breeding values, genomic selection, plant breeding, repeated record, time-dependent trait

## Abstract

The rapid development of remote sensing in agronomic research allows the dynamic nature of longitudinal traits to be adequately described, which may enhance the genetic improvement of crop efficiency. For traits such as light interception, biomass accumulation, and responses to stressors, the data generated by the various high-throughput phenotyping (HTP) methods requires adequate statistical techniques to evaluate phenotypic records throughout time. As a consequence, information about plant functioning and activation of genes, as well as the interaction of gene networks at different stages of plant development and in response to environmental stimulus can be exploited. In this review, we outline the current analytical approaches in quantitative genetics that are applied to longitudinal traits in crops throughout development, describe the advantages and pitfalls of each approach, and indicate future research directions and opportunities.

## Introduction

Enhancing agricultural production efficiency by reducing yield gaps while also breeding more stress-resilient cultivars is the next challenge for plant breeders (Godfray et al., 2010; Foley et al., 2011; Ray et al., 2013; Challinor et al., 2014; Tai et al., 2014). The most feasible solutions are developing innovative approaches to speed up the genetic improvement of economically important traits and characterizing novel traits and incorporating them into breeding programs (Duvick, 2005; Lange and Federizzi, 2009; Fischer and Edmeades, 2010; Nolan and Santos, 2012; Rogers et al., 2015).

Plant breeding was established as a science in the beginning of the 20th century, when new insights about the genetic basis of phenotypic variation for quantitative traits were integrated with the foundational theories of inheritance mechanisms and crop hybridization elucidated by Mendel and Darwin, respectively (Johannsen, 1909, 1911; East, 1911; Bradshaw, 2017). Since then, plant breeders have improved crop productivity by selecting for numerous traits. Breeding objectives are constantly refined to address new challenges, including adaptation to new production areas, addressing emerging pests and diseases, various end uses, advanced farming technologies, and climate change (Toenniessen, 2002; Baenziger et al., 2006; Tester and Langridge, 2010; Gilliham et al., 2017). For instance, in 1955 the focus of soybean breeding was to increase seed oil content, canopy ground cover, and ripening uniformity. Ten years later the focus shifted to reducing pod shattering and lodging, and then it changed again over the years to include quality and value-added traits (Baenziger et al., 2006). Similar shifts in breeding goals and phenotyping technologies have also occurred in animal breeding (Henryon et al., 2014; Miglior et al., 2017). However, throughout history, improving traits of interest depends on the ability to quantify phenotypes across genotypes replicated over multiple environments (Stoskopf et al., 1994). Therefore, potentially valuable traits may have been neglected due to costly phenotyping and technological limitations.

Plant phenotyping has always been paramount for genetic improvement. Recent advances in proximal remote sensing, paired with new sensors and computer science applications, has enabled cost-effective high-throughput phenotyping (HTP) and dissection of novel traits (Montes et al., 2007; Furbank and Tester, 2011; Fiorani and Schurr, 2013; Araus and Cairns, 2014; Coppens et al., 2017). HTP provides time-series measurements that track the development of a crop through its life stages and as it responds to the environment. Information on gene function, the activation of genes, interaction of genes networks at different stages of plant development and in response to environmental stimulus can now be exploited (Wu and Lin, 2006; Montes et al., 2007). It is increasingly possible for plant breeders to consider light interception, biomass accumulation, and response to drought stress as dynamic traits, rather than static points in time (Montes et al., 2007). This analytical framework enhances our understanding of crop development and bridges gaps in understanding the relationship between genotype and phenotype (Granier and Vile, 2014; Araus et al., 2018).

**FIGURE 1:**
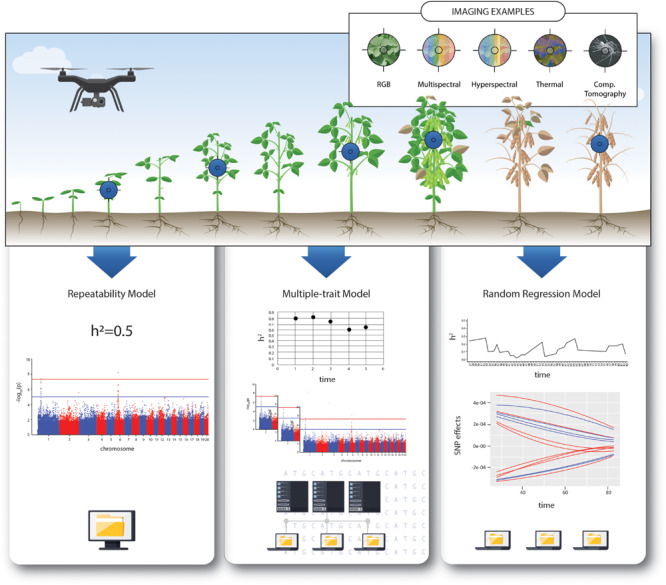
Schematic workflow of longitudinal data analyses. Different remote-sensing tools most commonly used for high-throughput phenotyping monitoring crop growth and development. Comparative overview of the potential models for genomic analysis, together with examples of outputs and computational demand.

Traits that are expressed repeatedly or continuously over the lifetime of an individual can be defined as longitudinal traits (Yang et al., 2006; Oliveira et al., 2019a), infinite-dimensional traits (Kirkpatrick and Heckman, 1989), or function-valued traits (Promislow et al., 1996). The study of longitudinal traits can provide important insights into the genetic mechanisms underlying physiological responses to environmental stresses and developmental processes. This information can be used to improve predictive ability for complex polygenic traits under multivariate settings, and contribute to identifying overall (e.g., soybean yield) or time specific Quantitative Trait Loci (QTL) (Fahlgren et al., 2015; Campbell et al., 2017; Sun et al., 2017). Such analysis enables assessment of the statistical association of genetic and environmental factors, such as the relationship between molecular markers and response to abiotic stress at different developmental stages (Langridge and Fleury, 2011). In this context, phenotypic data describes a function changing continuously in response to other variables (Stinchcombe and Kirkpatrick, 2012; Granier and Vile, 2014). These approaches generate a vast amount of data, which requires advanced statistical approaches to enable evaluation of the phenotypic data as a function of time. In this literature review, we will outline the current analytical approaches in quantitative genetics and genomics that can be applied to HTP quantified over time (Figure 1). In addition, we describe the advantages and pitfalls of each method and explore directions and opportunities for future research.

## Phenotyping Longitudinal Traits

Current HTP platforms, also referred to as “phenomic” tools, include a variety of methodologies that use remote sensing to obtain non-destructive phenotypic measurements, either in controlled environments or in the field (Pauli et al., 2016b). The most common types of sensors for crop phenotyping include red-green-blue (RGB; Xavier et al., 2017), multispectral (Xu et al., 2019), hyperspectral (Bodner et al., 2018), fluorescence (Pérez-Bueno et al., 2016), thermal (Sagan et al., 2019), three-dimensional (3D; Topp et al., 2013), and laser-imaging detection and ranging (LiDAR) (Sun et al., 2018) devices. In general, these sensors rely on the interaction between electromagnetic radiation and plants (reflecting, absorbing, or transmitting photons), which is captured by the sensor as reflected radiation (Fiorani et al., 2012; Li et al., 2014). Thus, the sensors interpret the plants as optical objects, with each component of the plant displaying a characteristic spectral signature arising from wavelength-specific properties of absorbance, reflectance, and transmittance from the vegetation surface (Schowengerdt, 2012; Li et al., 2014).

The spectral signatures of plants change during the life cycle, giving rise to genotype-time-specific phenotypes. For instance, during senescence, there is an increase of reflectance in the red region caused by a loss of chlorophyll (Schowengerdt, 2012). For field-based phenotyping, these sensing tools are usually integrated into ground or aerial vehicles (Araus et al., 2018). Most HTP platforms have the spatial and temporal resolution needed to capture longitudinal traits. However, the needs and resources of the specific experiment should drive the choice of platform and sensor, as these choices directly impact the scale and type of research (Pauli et al., 2016b). Several reviews have focused on HTP and its nuances, such as data collection, data processing and types of sensors (Fahlgren et al., 2015; Rahaman et al., 2015; Singh et al., 2016; Tardieu et al., 2017; Yang et al., 2017; Zhao et al., 2019; Reynolds et al., 2020).

The genetic control of longitudinal traits captured with HTP was recently reported for different crops. In a greenhouse, Neilson et al. (2015) investigated the growth and dynamic phenotypic responses of sorghum to water-limited conditions and various levels of fertilizer over time. They defined and measured several traits, including leaf area, shoot biomass, height, tiller number, and leaf greenness using laser scanning, RGB, and near-infrared (NIR) cameras. In barley, multiple sensors captured daily images in a greenhouse over 58 days measuring the spectrum of visible light, fluorescence, and NIR in order to dissect phenotypic components of drought responses (Chen et al., 2014). Tiller growth in rice was examined using more than 700 traits extracted from an imaging system combining computed tomography (CT) and RGB imaging during the tillering process (Wu et al., 2019).

In the field, Sun et al. (2018) used LiDAR mounted on a tractor to quantify leaf area, stem height, and plant volume in cotton in the field from 43 to 109 days after planting. From these measurements, they generated genotype-specific growth curves and assessed variability in traits and their genetic correlation to yield over time. In soybean, an unmanned aerial vehicle (UAV, aka drone) with an RGB camera measured canopy coverage in the field across several days after planting for genome-wide association analysis and genomic selection (Xavier et al., 2017; Hearst, 2019; Moreira et al., 2019). Blancon et al. (2019) characterized the dynamics of the green-leaf-area index in a diversity panel of maize under well-watered and water-deficient treatments using multispectral imagery acquired from a UAV throughout the growth cycle. Thermal and hyperspectral sensors mounted to a manned aircraft were used to extract canopy temperature and vegetation indexes of more than 500 lines of wheat in five field environments over a range of dates (Rutkoski et al., 2016).

Field-based HTP for roots has seen less progress than HTP of above-ground traits due to the difficulty of below-ground imaging (Atkinson et al., 2019). Shovelomics is a high-throughput method for root phenotyping and that has been used for crops, such as maize, common bean, cowpea, and wheat (Trachsel et al., 2011; Burridge et al., 2016; York et al., 2018). It consists of extracting several traits from images. However, it is destructive and labor-intensive, requiring manual root excavation, which limits its ability to capture repeated records over time. Recently, geophysical techniques, including electrical resistance tomography, electromagnetic inductance, and ground-penetrating radar have contributed to identify and quantify roots in the field in a non-destructive manner (Shanahan et al., 2015; Whalley et al., 2017; Liu et al., 2018a; Atkinson et al., 2019). Nevertheless, HTP root phenotyping is more commonly performed in controlled environments that allow the use of alternative growth systems that enable root imaging, such as rhizotrons, growth pouches and transparent artificial growth media (Atkinson et al., 2019; Ma et al., 2019). To model the growth dynamics of maize, Hund et al. (2009) performed daily scans of root systems grown over blotting paper. Topp et al. (2013) used 3D imaging phenotyping of rice root architecture in a gellan gum medium on various days of growth to perform QTL detection analysis. Recent advances in the use of X-ray CT and magnetic resonance imaging in plant sciences have enabled monitoring of root system architecture and dynamic growth over time in soil (Metzner et al., 2015; Pfeifer et al., 2015; van Dusschoten et al., 2016; Pflugfelder et al., 2017; Gao et al., 2019). More details and additional techniques for root phenotyping are presented in Atkinson et al. (2019).

## Modeling Longitudinal Traits

Plant growth and development are characterized by several phenotypic changes, which can only be studied by monitoring repeated phenotypes over time (Li and Sillanpää, 2015). HTP platforms allow tracking of traits with a high temporal resolution, whether continuously over time or in discrete intervals (Furbank and Tester, 2011). Traditionally, mathematical functions are used to describe temporal trajectories of traits during the plant’s life cycle (Paine et al., 2012). Analysis of longitudinal traits usually employs one of the following two techniques (Li and Sillanpää, 2013): (1) smooth functions (such as splines; e.g., van Eeuwijk et al., 2018; Oliveira et al., 2019a) or parametric functions (such as growth models; e.g., Paine et al., 2012) to fit the phenotypic records over time, providing interpolated values for all time points; or (2) the data is reparameterized by estimating the function’s coefficients, which are then used in the analysis to represent the trait over time. Either way, it is necessary to select the function that best fits the shape of the trajectory of the trait to accurately estimate the curve parameters and results. Paine et al. (2012) provide a detailed review about growth models, highlighting basic functional forms, advantages and disadvantages. In this section, we will describe the main functions that have been successfully used to fit a variety of traits for the purposes of crop improvement.

Many of the complexities of plant growth are commonly represented using non-linear growth models that account for temporal variation in growth, capturing age and size-dependent growth (Paine et al., 2012). Usually, the growth pattern within a plant life cycle follows a sigmoid curve (S-shaped) characterized by an initial slow growth that then increases rapidly, approaching an exponential growth rate, and finally slows when it reaches a saturation phase (Yin et al., 2003). The S-shaped curve can be described by sigmoidal functions such as the logistic, Gompertz, Richards or β functions (Gompertz, 1815; Richards, 1959; Yin et al., 2003; Poorter et al., 2013). In this case, the Gompertz function is a special case of the Richard function; which is one of the oldest growth models frequently used to fit various biological processes across species (Tjørve and Tjørve, 2017). The Gompertz function has been used to describe biomass accumulation in maize kernels (Meade et al., 2013), barley biomass (Chen et al., 2014), and various longitudinal traits in wheat (Camargo et al., 2018) and sorghum (Neilson et al., 2015). The Logistic function is more commonly used in its asymptotic form to describe the time dependence of biological growth processes for traits such as biomass, canopy coverage, canopy size, volume, length, and area (Thornley et al., 2005; Paine et al., 2012). The Logistic function can have one, two, three, four, or five-parameters (Tessmer et al., 2013). One- and two-parameter logistic models are simplistic and frequently do not fit the data well, but are still used in several studies (e.g., Paine et al., 2012; Tessmer et al., 2013). The three-parameter logistic function (3PL; also known as a Verhulst or autocatalytic growth function) is perhaps the most popular model for plant growth analysis. In a water-limitation experiment in sorghum, a 3PL model had the best performance of a variety of sigmoidal models to fit the projected leaf area (Neilson et al., 2015). Sun et al. (2018) used a 3PL model to fit growth curves for canopy height, projected canopy area, and plant volume obtained from HTP in cotton. In wheat, Baillot et al. (2018) estimated various grain-filling parameters by fitting a 3PL model. The four-parameter logistic (4PL) model is more flexible than the 3PL as it has fewer constraints (Pinheiro and Bates, 2000). Camargo et al. (2018) phenotyped the average of area, height, and senescence in wheat throughout its lifecycle and found that 4PL models best fit the longitudinal data. The five-parameter version (5PL) provides maximum flexibility and accommodates asymmetry (Gottschalk and Dunn, 2005), despite its higher complexity compared to the lower number of parameters.

Many biological curves cannot be described by sigmoidal functions. A Power Law (also known as allometry) function is a type of non-asymptotic, non-linear growth model that does not produce an S-shaped curve (Marquet et al., 2005). They are often used in ecology to predict relationships in plant communities (Chen and Shiyomi, 2019). It effectively captures temporal variation in growth as it allows the relative growth rate to slow down over time and with an increase in biomass (Paine et al., 2012). A Power Law function was used to fit projected leaf area data in sorghum receiving various levels of nitrogen (Neilson et al., 2015), as well as leaf length and rosette area in *Arabidopsis thaliana* (An et al., 2016).

Linear models, such as orthogonal polynomials and spline functions, are also used to fit longitudinal traits (Oliveira et al., 2019a). The use of polynomials in crop growth models started in the 1960s as a functional approach to fit growth data and provide a clear picture of ontogenetic drift (Vernon and Allison, 1963; Hughes and Freeman, 1967; Poorter, 1989). One of the advantages of these functions is that they do not require prior knowledge of the longitudinal shape of the phenotype. Therefore, they are useful for fitting biological data of any shape simply by choosing different orders of the polynomials. Although they are not linear in time, polynomial functions are linear in their parameters, and consequently, can take advantage of the inference methods available for linear models (Yang et al., 2006). For instance, cubic polynomial functions have been used to describe grain growth in crops such as rice (Jones et al., 1979; Shi et al., 2015), wheat (Gebeyehou et al., 1982), barley (Leon and Geisler, 1994), and safflower (Koutroubas and Papakosta, 2010). One of the main difficulties with this approach is choosing the appropriate degree of polynomial to fit the data while avoiding spurious upward or downward trends or overfitting or underfitting the data (Paine et al., 2012).

Orthogonal polynomials are particularly popular for fitting biological curves because they have much lower correlations among their coefficients and provide estimates of the covariance matrices that tend to be more robust over a variety of data sets (Schaeffer, 2004). Legendre polynomials represent simple orthogonal polynomials and have been used successfully to fit longitudinal traits in livestock breeding programs (e.g., Albuquerque and Meyer, 2001; Oliveira et al., 2017, 2019b; Brito et al., 2017) and for plant research (e.g., Yang et al., 2006; Yang and Xu, 2007; Campbell et al., 2018; Momen et al., 2019).

Spline functions offer a more flexible alternative for modeling longitudinal traits compared to orthogonal polynomials (van Eeuwijk et al., 2018). Splines are piecewise polynomial functions, linked at specific points called knots (de Boor, 1980). For longitudinal data, these knots represent time points within the data collection interval (Li and Sillanpää, 2015). The greater flexibility of splines is due to the independence of each segment, which can have the same or different polynomial degrees, accommodating abrupt changes in the trajectory (Meyer, 2005b). A particular type of spline function is the basis spline, or B-spline (de Boor, 1980), extensively deployed in animal breeding (Meyer, 2005b; Oliveira et al., 2019a). Another version of spline is P-spline, which combines B-splines with different penalties on the coefficients of adjacent B-splines, resulting in smoother curves (Eilers and Marx, 1996; Meyer, 2005b).

Spline functions have recently been used to model longitudinal traits in crops. For instance, haulm senescence was assessed at several points during the growing season in a diploid potato mapping population and fitted using P-splines (Hurtado et al., 2012). Montesinos-López et al. (2017) used a B-spline function to fit wheat canopy hyperspectral bands in a yield prediction model. B-splines have also modeled rice temporal shoot biomass in a water-limited environment (Momen et al., 2019).

## Statistical Genetic Models

Plant breeding is mostly based on the selection of new genetically superior cultivars from a large set of candidates. Simple arithmetic means of the phenotypic values, or Best Linear Unbiased Estimation (BLUE, treating genotypes as fixed effects), were used for selection prior to the development of Best Linear Unbiased Prediction (BLUP; Henderson, 1974). BLUPs are based on a mixed linear model and are now the most commonly used method for genetic evaluation of plant and livestock species (Piepho et al., 2008; Mrode, 2014). In the mixed-model framework, the genotypes are fitted as random and the genotypic effects are estimated by BLUP. The main advantage of BLUP over previous methods is its increased prediction accuracy for genetic effects. This is due to the shrinkage toward the mean that depends on the amount of information available (from the individual and/or its relatives), which will adjust extreme high and low performance toward the overall mean, and also to the incorporation of the genetic correlation between related genotypes from pedigree or genomic information (Piepho et al., 2008). The latter is not a requirement for the model, so the simplest case of BLUP uses no relationship matrix and the genotypes are considered to be independent random variables (Yan and Rajcan, 2003; Cullis et al., 2006). Piepho et al. (2008) present several examples of BLUP analyses in plant breeding.

Although rarely used, pedigree data is an easy and inexpensive source of information for plant breeders to leverage the relationship between individuals for a more accurate estimation of breeding values. Pedigree-based BLUPs have been successfully used in various crops (Bromley et al., 2000; Rutkoski et al., 2016; Basnet et al., 2019; Moreira et al., 2019), and have contributed to major advancements in the rates of genetic progress.

The inclusion of genomic information provides more accurate estimates of genetic relatedness among genotypes, especially with regards to the Mendelian sampling effects (Habier et al., 2007). Genomic information traces allele inheritance, capturing small segments of the genome shared among individuals, even when they are apparently unrelated through pedigree (Velazco et al., 2019). Plant breeders have widely adopted genomic-based BLUPs (GBLUP) for genomic selection (Auinger et al., 2016; Crossa et al., 2017; Schrag et al., 2019). Although genomic information is promising, in practice high-density genotyping is not always feasible for all genotypes within a breeding program due to genotyping costs, logistics, or both (Habier et al., 2009). An alternative is to construct a joint relationship matrix based on pedigree and genomic relationships to predict BLUPs for genotyped and non-genotyped material, which is called single-step GBLUP (ssGBLUP; Misztal et al., 2009; Aguilar et al., 2010; Christensen and Lund, 2010). This approach integrates both relationship matrices, connecting their different yet complementary information on genetic relatedness, and provides more reliable and accurate estimates of genetic similarities between genotypes. Genomic breeding values based on the ssGBLUP approach are commonly used in animal breeding (Aguilar et al., 2010; Legarra et al., 2014; Meuwissen et al., 2015; Guarini et al., 2019a, b; Oliveira et al., 2019c), and their use has started to become popular in plant breeding as well (Ashraf et al., 2016; Cappa et al., 2019; Velazco et al., 2019). In sorghum, Velazco et al. (2019) demonstrated that this methodology improves the predictive ability for complex traits, especially for traits with low heritability estimates, measured late in the development stage, or those that are difficult or expensive to measure.

For longitudinal traits, one can calculate BLUPs for each time point separately as individual traits with unique phenotypes; however, these approaches do not directly investigate and compare trends over time (Littell et al., 1998). This makes it difficult to consider a large number of time points and inhibit data comparison when BLUPs shrink differently due to discrepancies in heritability estimates. The main goal of fitting curves and patterns for longitudinal traits is to consider variability in the developmental process across many points in time (e.g., growth). Analytical methods have been developed to better evaluate longitudinal traits using the BLUP context, a simple analysis of variance, or both (Littell, 1990; Meyer and Kirkpatrick, 2005; Mrode, 2014). We will discuss the main methods in this review.

### Repeatability Model

Individual measurements recorded over time can be treated as repeated records of the same trait. This is known as the repeatability model. There are two critical assumptions implicit in this method: (1) the variances of different measurements within the same genotype (or individual) are always equal, regardless of the time interval between records; and (2) the genetic correlations between all measurements are equal to one, i.e., measurements at different time points are all influenced by the same genes (Falconer and Mackay, 1996; Meyer and Hill, 1997; Littell et al., 1998). In this scenario, simple repeatability models are the standard approach.

One of the simplest methods is the repeated-measurements analysis of variance (ANOVA) using a split-plot in time design, which treats the genotypes as a whole-plot unit and genotypes at particular times as a sub-plot unit (Rowell and Walters, 1976; Littell, 1990). It is important to mention that as time is a factor in the experiment that cannot be randomized, this is not a true split-plot design. Also, this method assumes the data have equal variances (homoscedasticity) in all repeated measurements and that all pairs of measurements will have the same correlation (i.e., compound symmetry), which are unrealistic assumptions for most crop datasets. However, Huynh and Feldt (1970) showed that the equality of the variances of differences between any two treatment measurements assumed to be correlated was sufficient to perform a split-plot ANOVA. In this case, if the data violate the Huynh and Feldt condition, the F-statistics for the sub-plot unit and their interaction will be inflated. Thus, this method is prone to high Type I error rates, leading to conclusions that effects are statistically significant when they are not (Scheiner and Gurevitch, 2001; Fernandez, 2019).

In the context of mixed models, specifying the random and fixed effects in the model will depend on the study objectives, data structure, and the assumptions that can be made. Usually, time is considered as a fixed effect because it is not randomized in an experiment. Simple repeatability models have been used to calculate BLUPs and BLUEs of longitudinal traits derived from HTP for genomic prediction, such as in wheat (Rutkoski et al., 2016; Sun et al., 2017).

### Multiple-Trait Model

Often, HTP platforms are used to generate phenotypes of plants in different “ages” or development stages, with the mean and variance of the phenotypes between measurements/assay dates changing over time. Thus, the assumption is that the genetic control of longitudinal traits will be different over time, characterizing the longitudinal records/phenotypes as different traits. A common approach to analyze longitudinal traits in this scenario is a multi-trait analysis that considers each time point as a different dependent variable (Sun et al., 2017).

Multivariate analysis of variance (MANOVA) is an extension of ANOVA, mentioned earlier, that avoids the covariance structure problems raised in repeated-measures ANOVA. However, it still requires equality in covariance among the groups being compared and balanced data over time. In addition, MANOVA assumes a multivariate normal distribution. Alternative methods have been proposed to overcome these restrictions (Krishnamoorthy and Lu, 2010; Krishnamoorthy and Yu, 2012; Konietschke et al., 2015), but MANOVA still has limited use in practice.

In the BLUP context, multiple-trait mixed models were first implemented by Henderson and Quaas (1976) to analyze two or more correlated traits making use of genetic and residual covariances among the traits (Speidel, 2011). Using this method, one can directly model the covariance structure of multiple dependent variables and efficiently handle missing data (Mrode, 2014). The main advantage of using a multiple-trait model (MTM) over a single-trait repeatability model is the improved evaluation accuracy for each trait arising from better connections in the data between the genetic and residual covariance (Colleau et al., 1999; Mrode, 2014). This data structure will benefit the prediction of traits with lower heritabilities when combined with highly heritable traits and genotypes with missing records for one or more traits (Mrode, 2014). In wheat, MTM was used to predict BLUPs for canopy temperature and normalized difference vegetation index (NDVI; Sun et al., 2017), and BLUEs for green NDVI (Juliana et al., 2018).

There are some disadvantages of multiple-trait mixed models. For instance, high-dimensional longitudinal data (e.g., traits recorded multiple times over a long period) can lead to over-parameterized models with high computational requirements (Speidel, 2011). There is also the potential for high correlations between consecutive measurements, which can reduce the power of the tests of significance (Foster et al., 2006). There are approaches to reduce the dimensionality of the MTM, which we are discussed below. It is worth noting that, when applying these approaches, the appropriate models should still be adequate to describe the data, accounting for the changes of mean and covariance over time, and estimate the necessary genetic parameters (Mrode, 2014).

Canonical transformation of phenotypic records is a common procedure to eliminate autocorrelation among traits through eigenvalue decomposition (Meyer and Hill, 1997). A set of highly correlated measures will provide eigenvalues close to zero. Under the framework of canonical transformed phenotypes, the original observations are transformed into a new set of response variables and the ones with the highest eigenvalues are selected to compose the new combination of traits. After fitting the MTM with the new values, the results are transformed back to the original scale (Mrode, 2014). Grosu et al. (2013) highlighted that canonical transformation can only be used if all individuals are recorded for all the traits and that the model needs to be the same for each trait, accommodating only two random effects: residual and genetic. Another strategy to fit MTM is referred to as “bending” (Thompson and Meyer, 1986; Meyer, 2019). It does not require all traits to be measured in all individuals. This procedure forces a decreased autocorrelation among traits by shrinking the covariance among traits by a bending factor, which creates a positive-definite covariance matrix.

The principal component analysis (PCA) and factor analysis (FA) methods are often more appropriate to reduce dimensionality for a large number of traits. FA identifies common factors, called latent variables, associated with the correlations between variables (Mrode, 2014). On the other hand, the PCA approach aims to create independent variables (principal components) that explain the maximum amount of variation in the dataset (Mrode, 2014). Thereafter, the principal components or latent variables become the new dependent variables in the MTM. Both methods have been used to reduce the dimensionality of longitudinal trait analysis in animals (Macciotta et al., 2017; Durón-Benítez et al., 2018; Vargas et al., 2018), and plants (Kwak et al., 2016; Yano et al., 2019).

As longitudinal traits are, by definition, taken along a time trajectory, the whole data set can be represented by parameters describing the shape of the trajectory curve (e.g., growth curve). These parameters can become the new dependent variables or integrated covariance structures in MTM (Speidel, 2011; Oliveira et al., 2019a); however, none of the approaches to analyze longitudinal data that we have discussed so far have considered that the genetic and environmental variances may change over time (Meyer, 1998, 2005a; Oliveira et al., 2019a). In addition, these approaches are limited to the time points at which traits were measured. Random regression models (RRMs) provide a way to overcome these limitations (Schaeffer, 2004).

### Random Regression Model

A common property of longitudinal traits is that the covariance between repeated measures depends on the interval of time between them. In other words, measurements collected closer in time will be more correlated than measures collected farther apart. Kirkpatrick et al. (1990) presented the concept of analyzing longitudinal data using covariance functions by describing the covariance structure of the traits as a function of time. In essence, this approach fits a set of orthogonal functions to a given covariance matrix for the records taken over time (Meyer and Hill, 1997).

First-order autoregressive analysis (AR-1) is an appealing method for modeling covariance structure for phenotypes measured over time (Apiolaza and Garrick, 2001; Yang et al., 2006; Vanhatalo et al., 2019). It assumes homogenous variances and correlations that decline exponentially as measurements are separated by greater time intervals. Thus, two measurements collected closer in time will be more correlated than those further apart (Wade et al., 1993; Littell et al., 2000; Piepho et al., 2004). The AR-1 structure is only applicable for measurements taken at equally spaced time points (Wang and Goonewardene, 2004). Though this is a difficult requirement to meet in agricultural research, especially in field trials, modeling the longitudinal trait as described in the previous section would make the data evenly spaced over time and validate the AR-1 method. An alternative is to use a spatial power covariance structure that allows for unequal intervals between time points (Wang and Goonewardene, 2004).

Note that so far, we are assuming homogenous variance over time. There are also covariance structures to handle heterogeneous variance, such as the first-order ante-dependence structure (Wolfinger, 1996). Thus, Legendre orthogonal polynomials and splines are more attractive covariance functions as they produce relatively small correlations among the regression parameters and adjust flexibly to the shape of the trajectory curve (Schaeffer, 2004; Meyer, 2005a, b; Bohmanova et al., 2008; Pereira et al., 2013; Brito et al., 2018). In plants, different covariance structures have been assessed for a variety of traits (Apiolaza et al., 2011; Sun et al., 2017; Campbell et al., 2019).

Meyer and Hill (1997) showed that covariance functions are equivalent to RRMs. Schaeffer (2016) reported that covariance functions help to predict the change in variation over time, while RRMs are a way to estimate covariance functions and determine individual differences in trajectories. RRMs provide a robust framework for modeling trait trajectories using covariance at or between each time point with no assumptions of constant variances or correlations. RRMs provide insights about the temporal genetic variation of developmental behavior underlying the studied traits (Oliveira et al., 2019a). Despite the increased computational cost, RRMs result in more accurate breeding values compared to other methods (Sun et al., 2017; Oliveira et al., 2019a).

The RRMs were first introduced in animal breeding to overcome over-parameterized models in MTM and they have been used extensively since then (Jamrozik and Schaeffer, 1997; Schaeffer, 2004; van Pelt et al., 2015; Englishby et al., 2016; Oliveira et al., 2019a). In summary, RRMs set the parameters of the function describing the trajectory of the trait as fixed and random effects in the model, resulting in fewer parameters than MTM (Schaeffer, 2016; Oliveira et al., 2019a). Consequently, in RRMs the random parameters do not correspond directly to the individuals’ genetic value for the longitudinal trait. Rather, they correspond to the genetic values of sets of regression coefficients that represent the time trajectory of the longitudinal trait for each genotype (Turra et al., 2012). Estimates of genetic parameters and breeding values can be obtained for all time points within the interval analyzed from the genetic (co)variance matrices for the regression coefficients and the matrix of independent covariates for all time points associated with the function used (Oliveira et al., 2019a). When the same fixed effects are used in all models, it is appropriate to examine different covariance structures using real data and select the one that best fits the model based on a statistical methods, such as the Akaike Information Criterion (AIC, Wang and Goonewardene, 2004) or Bayesian Information Criterion (BIC, Neath and Cavanaugh, 2012). Finally, estimate the effects of interest using the selected covariance structure. In a general form, RRM can be described as follows (Oliveira et al., 2019a):

$${\text{y}}_{\text{gij}}=\sum_{\text{q}=1}^{\text{Q}}{\text{b}}_{\text{qg}}\,{\text{z}}_{\text{qg}}+\sum_{\text{r}=1}^{\text{R}}{\text{a}}_{\text{ri}}\,{\text{z}}_{\text{ri}}+\sum_{\text{s}=1}^{\text{S}}{\text{p}}_{\text{si}}\,{\text{z}}_{\text{si}}+{\text{e}}_{\text{gij}}$$

where y_gij_ is the jth repeated record of genotype i (e.g., canopy coverage at different days after planting); b_qg_ is the qth fixed regression coefficient for the gth group; a_ri_ is the rth random regression coefficient for the additive genetic effect of the ith genotype; p_si_ is the sth random regression coefficient for permanent environmental effect of the ith genotype; e_gij_ is the residual effect; and z_qg_, z_ri_ and z_si_ are the covariates related to the function used to describe time (e.g., days after planting), assuming the same function (e.g., Legendre polynomial) with possible different orders Q, R, and S (e.g., linear, quadratic, cubic) (Oliveira et al., 2019a).

Random regression models have been shown to be the most effective choice to genetically evaluate longitudinal traits in numerous livestock breeding programs (as reviewed by Oliveira et al., 2019a), but there are only a few examples of the applications of RRMs in plant breeding, especially when incorporating genomic information. Sun et al. (2017) captured the change of HTP traits continually over wheat growth stages using RRMs. Campbell et al. (2018) used RRMs to predict shoot growth trajectories in a rice diversity panel and demonstrated an improvement in prediction accuracy compared to a single-time-point model. Based on the same rice dataset, Campbell et al. (2019) used RRMs to identify QTL with time-specific effects. Multiple-trait RRMs are also feasible and have been implemented in several settings in animal breeding programs (Nobre et al., 2003; Muir et al., 2007; Oliveira et al., 2019b, c).

## Implementation of Genomic Selection for Longitudinal Traits

Meuwissen et al. (2001) introduced the concept of genomic selection (GS) based on the idea that markers from dense genome-wide genotyping will be in linkage disequilibrium with QTLs that have an effect on the quantitative trait of interest. Thus, they can be used for selection without identifying the QTL or the functional polymorphism. This increased understanding of GS arose as it became known that markers would carry relationship information in addition to the signal captured by the linkage disequilibrium between markers and QTL (Habier et al., 2007; Meuwissen, 2009).

In GS, genomic and phenotypic data are combined in a training population to enable the development of prediction equations that can be used in a testing (or target) population of selection candidates consisting of individuals that were genotyped but not phenotyped (Crossa et al., 2017). Therefore, GS enables a more accurate selection of individuals at an early age (with no measurements). This increases the rate of genetic gain by reducing the time required for the variety development and the cost per cycle. HTP is able to generate high-quality quantitative data and effectively characterizes large training populations during the growing season. The combination of GS and HTP has the potential to increase precision and efficiency while lowering costs and minimizing labor (Araus et al., 2018).

Under the longitudinal framework of GS, the prediction of temporal breeding values enables targeted selection on specific periods in the growing season or selection of individuals that exhibit desirable trait trajectories. In addition, the longitudinal trait can be used as secondary traits to improve the genomic selection of economic endpoint traits such as yields (Sun et al., 2017). Campbell et al. (2018) used RRMs with a second-order Legendre polynomial to perform pedigree and genomic predictions of shoot growth trajectories in a rice diversity panel. They demonstrated an improvement in prediction accuracy using the RRM compared to a single-time-point model. Furthermore, the authors reported that genomic RRMs were useful in predicting future phenotypes using a subset of early measurements. Another study in rice used RRMs to predict projected shoot area in controlled and water-limited conditions using Legendre polynomials and B-spline basis functions (Momen et al., 2019). Before fitting both functions, they adjusted raw phenotypic measurements to obtain BLUEs for downstream genetic analysis. Overall, RRMs produced higher prediction accuracy compared to the baseline multiple-trait model. In addition, B-splines performed slightly better than Legendre polynomials (Momen et al., 2019).

Currently, statistical models used in GS for plant breeding are most often single-trait (univariate) and do not take advantage of genetic covariance among traits or phenotypic records collected at different time points (Jia et al., 2012). However, MTM for GS was shown to outperform single-trait models by accounting for correlation among traits, thereby increasing prediction accuracy, statistical power, parameter estimation accuracy, and reducing trait selection bias (Jia et al., 2012; Guo G. et al., 2014; Montesinos-López et al., 2016, 2019). These advantages are even more obvious for low-heritability traits, such as yield, that are genetically correlated with highly heritable traits (Guo G. et al., 2014; Jiang et al., 2015). Recently, studies in the CIMMYT (2019) wheat breeding program^1^ have shown that the accuracy of GS is greatly improved by incorporating HTP longitudinal data from the so-called secondary traits measured with UAV (Rutkoski et al., 2016; Montesinos-López et al., 2017; Sun et al., 2017, 2019), an approach that is relatively inexpensive to implement as HTP and genotyping have become more accessible (e.g., targeted genotyping-by-sequencing approach; Pembleton et al., 2016). In addition, secondary traits are also useful to predict the primary trait at early growth stages, since they can often be phenotyped ahead of a primary trait like grain yield (Sun et al., 2017). Therefore, longitudinal traits can be used as secondary traits to improve the accuracy of GS and contribute to a better understanding of the biological mechanisms underlying stress responses and development. As described in the previous section, there are various ways to extract the genetic information from longitudinal traits and the methods employed will determine how they can be used in GS.

Rutkoski et al. (2016) used HTP canopy temperature (CT), green normalized difference vegetation index (GNDVI), and red normalized difference vegetation index (RNDVI) taken over time as secondary traits in GS for yield in wheat. First, they estimated BLUEs for the longitudinal traits using the repeatability model and used them in an MTM with yield, for pedigree and genomic predictions. They found that multiple-trait modeling with secondary traits increased accuracies for grain yield using both pedigree and genomic information, compared to the single-trait models. In another study, CT and NDVI also improved the ability to predict grain yield in wheat (Sun et al., 2017). However, in addition to a repeatability model, the authors also used MTM and RRMs to calculate BLUPs for the secondary traits in order to compare their efficiency. The predictive ability improved by 70%, on average, when including secondary traits, and the predictive ability of RRM and MTM were superior to the repeatability model. Also in wheat, Juliana et al. (2018) performed pedigree and genomic multi-trait prediction models using BLUEs of yield and GNDVI measured at different dates. They found that including GNDVI increased prediction accuracies. Sun et al. (2019) used an RRM with a cubic smoothing spline to predict BLUPs for CT and GNDVI in wheat. In a second step, they used BLUPs for secondary traits and grain yield as the dependent variables in GS. The prediction accuracy using the secondary traits increased by an average of 146% for grain yield across cycles and the secondary traits measured in the early stages were optimal for enhancing the prediction accuracy. Montesinos-López et al. (2017) and Crain et al. (2018) obtained similar results in wheat. Howard and Jarquin (2019) modeled the genetic covariance between canopy coverage and yield using the SoyNAM dataset (Song et al., 2017; Diers et al., 2018) and demonstrated that, based on different cross-validation schemes, the predictive ability was the highest when both canopy and marker information were included in the model. Two other papers reported similar improvements with the same dataset (Xavier et al., 2017; Jarquin et al., 2018).

Given the capability of HTP to collect multiple temporal traits at the same time, multiple-trait RRMs (MTRRMs) can be powerful tools for joint genomic prediction of several longitudinal traits (Oliveira et al., 2016). In addition, MTRRMs can incorporate different functions to describe different traits in the same model and estimate genetic correlations between different traits over time (Oliveira et al., 2016). In animals, MTRRMs are a plausible alternative for joint genetic prediction of milk yield and milk constituents in goats (Oliveira et al., 2016), cattle (Oliveira et al., 2019c), and buffaloes (Borquis et al., 2013). Recently, MTRRMs for projected shoot area and water-use recorded daily over a period of 20 days showed better predictive abilities compared to single-trait RRMs (Baba et al., 2020).

In animal breeding and multiple-stage plant breeding analysis, it is common to use deregressed genetic values as the pseudo-phenotypes for genomic predictions. Oliveira et al. (2018) compared different deregression methods for longitudinal traits. However, this multiple-step approach may result in lower accuracies, bias, and loss of information (Legarra et al., 2009; Kang et al., 2017). Considering the advantages of ssGBLUP and RRMs in genetic evaluation, integrating both approaches is an effective strategy to enhance the genomic prediction of longitudinal traits (Kang et al., 2017). Koivula et al. (2015) reported higher accuracy and less bias in the prediction of Nordic Red Dairy cows for milking performance using a ssGBLUP RRM compared to the traditional pedigree-based RRM. Kang et al. (2017) showed that ssGBLUP RRMs achieved the highest accuracy and least bias under a variety of scenarios, including persistency of accuracy over generations, compared to other models. In summary, the use of ssGBLUP based on RRMs can increase the reliability of genomic predictions for test-day traits in dairy cattle (Koivula et al., 2015; Kang et al., 2018; Oliveira et al., 2019c), and possibly in crops.

## Detecting QTL and Causal Variants Associated With Longitudinal Traits

One of the main goals in genomic research is to predict the phenotypic variation using genotypes, by identifying genetic variants. The development of an organism is the result of an interacting network of genes and environmental factors (Wu and Lin, 2006). Unlike single-time-point measurements, studying longitudinal traits as a function of time allows the comprehensive assessment of crop growth and development (e.g., age metabolic rate; Ma et al., 2002). However, in plants, the detection of QTL analysis or genome-wide association studies (GWAS) for longitudinal traits are still performed at each time point independently. For instance, Würschum et al. (2014) used linkage mapping at discrete time points separately to identify time-specific QTLs associated with plant height in triticale. In cotton, canopy-related traits were used separately for each of the several studied days to map additive QTL effects and their interaction with the environment (Pauli et al., 2016a). In soybeans, a GWAS was used to identify QTL for each individual canopy coverage measurement spanning 14–56 days after planting (Xavier et al., 2017). Zhang et al. (2017) performed QTL mapping for several growth-related traits at 16 time points separately in maize. Also in maize, analysis of individual time points found different and simultaneous QTLs controlling plant height at different growth stages (Wang et al., 2019). Using time point growth-related traits, Knoch et al. (2020) found evidence of temporal QTLs in canola. Although useful, these static examinations provide a simplified view of genetic control, neglecting temporal changes and developmental features of trait formation (Wu and Lin, 2006). In addition, in animals, it has been shown that neither the phenotypic nor additive polygenic effects of longitudinal traits are constant throughout the entire phenotypic expression (Szyda et al., 2014; Brito et al., 2018; Oliveira et al., 2019a).

As an alternative, Ma et al. (2002) proposed a dynamic model, called functional mapping, to map QTLs associated with the whole developmental process of longitudinal traits. As mentioned earlier, longitudinal traits can be represented as curves, described by a few parameters from a linear or non-linear function over a given time. The idea behind functional mapping is that the difference in curve parameters among genotypes may suggest temporal patterns of genetic control over the phenotypic trajectory (Ma et al., 2002). Thus, functional mapping allows testing of timing and the duration of QTL expression (Wu et al., 2004). Several modeling strategies for functional mapping have been proposed and have been reviewed by Li and Sillanpää (2015). One of the approaches (the two-stage method) consists of modeling the whole phenotypic trajectory using linear and non-linear models and using these parameters as latent-trait phenotypes for QTL detection (Li and Sillanpää, 2015). Often, researchers perform analyses for individual time points followed by this two-stage method to derive the curve parameters. Busemeyer et al. (2013) used a logistic function to fit high-throughput-derived biomass from different development stages of a large mapping population of 647 double-haploid triticale lines. In addition to GWAS for the individual days, they performed a multiple-trait functional GWAS using the parameters from the logistic curve to reveal temporal genetic patterns of biomass regulation. A similar approach was used to assess image-derived biovolume in maize lines (Muraya et al., 2017); digital biomass accumulation in spring barley (Neumann et al., 2017); and area, height, and senescence in wheat (Camargo et al., 2018). Campbell et al. (2017) calculated the projected shoot area in 360 rice accessions from 19 to 41 days after transplanting. They modeled the longitudinal phenotypes using a power function (Paine et al., 2012) and used the parameters as the pseudo-phenotypes in a multiple-trait GWAS. In order to reveal the temporal dynamics of senescence in potato, Hurtado et al. (2012) employed P-splines as a smoothing curve and used the curve parameters for identifying QTLs.

Kwak et al. (2014) proposed two simple regression-based methods to map QTL by analyzing each time point separately and then combining test statistics across time points to determine the overall significance. Later, Kwak et al. (2016) proposed an improved approach where the observed longitudinal traits were replaced by a smoothing approximation, followed by dimensional reduction via PCA. Multiple-trait QTL analysis was then performed on the reduced data (using principal components). Muraya et al. (2017) implemented the approach suggested by Kwak et al. (2016). They used B-splines to smooth the phenotype followed by PCA for variable reduction and performed a multiple−QTL analysis following the methods of Kwak et al. (2014) to reveal the underlying genetic variation of growth dynamics in maize. Temporal height QTLs in the model C4 grass *Setaria* were also revealed using this approach (Feldman et al., 2017). Animal breeders have been using PCA for longitudinal trait analysis for a long time to synthesize complex patterns and reduce computationally demanding multiple-trait QTL detection (Macciotta et al., 2006, 2015, 2017; Zhang et al., 2018).

RRMs offer a better option to fit longitudinal traits and have been widely used in genetic evaluation of animals (Ning et al., 2017; Oliveira et al., 2019a). The random regression approach uncovers SNP effects over time because it is able to identify persistent and time-specific transient QTLs. Moreover, RRMs have increased statistical power to detect QTLs over other approaches because they leverage the full set of raw longitudinal phenotypes (Ning et al., 2017) and can capture QTLs with significant effects in specific regions of the development curve, though the effects of these QTLs may be small overall. RRMs are also useful for detecting QTL in gene-by-environment interactions (Lillehammer et al., 2007; Carvalheiro et al., 2019).

Das et al. (2011) proposed a method called functional GWAS (*f*GWAS), based on RRMs, which integrates GWAS and mathematical models describing biological processes. In summary, *f*GWAS estimates the mean for different SNP effects for each genotype and time point and then performs hypothesis testing to determine whether the SNP has any additive or dominant effect during the time course. The main drawback of this method is that it only performs a single-locus analysis. Later, Ning et al. (2017) proposed a modification of *f*GWAS by estimating the time-dependent population mean and the SNP effects separately, instead of fitting them directly. They also extended the model to capture the time-varying polygenic effect of complex traits by treating SNPs as covariates (*f*GWAS-C) or factors (*f*GWAS-F). However, their method was shown to be computationally inefficient due to the high dimensionality of the mixed model equations compared to other models. Subsequently, Ning et al. (2018) proposed a rapid longitudinal GWAS method, transforming the covariance matrices to diagonal matrices using eigen-decomposition. This way the model can be solved by a weighted least squares model for each SNP test.

To the best of our knowledge, Campbell et al. (2019) were the first to use RRM GWAS in a major row crop. They took the genomic breeding values derived from RRMs using Legendre orthogonal polynomials to assess the genetic architecture of rice shoot growth over a period of 20 days during early vegetative growth. They found both transient and persistent effects associated with shoot growth and more associations with the RRM when compared to single-time-point analysis.

## Challenges and Future Developments

To capitalize on advances in phenotyping and molecular technologies, greater progress is needed in developing ways in which breeders can manipulate systems to understand the relationships between genotype and phenotype. The underlying biological changes due to environmental systems and/or over time can be captured with longitudinal data. The major challenge is synthesizing the various layers of information together in a meaningful manner to understand the downstream effects of developmental stress and implications for breeding (Harfouche et al., 2019).

### Non-Additive Effects and GxE

Non-additive genetic effects may significantly contribute to the total genetic variation of complex traits. Prediction models that include dominance effects represent an important component of breeding programs that focus on crossbred populations, hybrid production, and vegetatively-propagated species (de Almeida Filho et al., 2019). There is also ample evidence of the importance of epistasis in the genetic architecture of complex traits in various crops (Guo T. et al., 2014; Monir and Zhu, 2018). Integrating non-additive effects into statistical models may improve prediction accuracy and detect more QTLs than simple additive models, especially when the non-additive variance contributes to a large proportion of the genetic variance (Bouvet et al., 2016; Bonnafous et al., 2018; Liu et al., 2018b, 2019; Monir and Zhu, 2018; Varona et al., 2018). However, these studies are restricted to single-time-point traits. For longitudinal traits, it may be challenging to have a full genetic model (including both additive and non-additive effects), requiring dense marker panels to estimate the time-dependent (co)variances, as well as partitioning of the genetic variance components. Nevertheless, full genetic models of longitudinal traits may have the potential to impact future design and implementation of breeding strategies.

The temporal dynamics of longitudinal traits lead to interactions that change the phenotype over time. This may be because the gene-gene and gene-environment interactions (GxE) are time- or age-dependent and need to be properly modeled (Fan et al., 2012). In this case, environmental descriptors should be measured several times as the trait phenotypes. The resulting model is a multiple-trait, multiple-environment model with a variety of interactions, in which computational issues may arise due to the increase in the number of parameters being estimated. It has been shown that RRMs can account simultaneously for the additive genetic effect and some degree of GxE in longitudinal traits in animal breeding by allowing for the estimation of genetic (co)variance components and breeding values over the whole trajectory of a time-dependent trait and environment-dependent covariate (Brügemann et al., 2011; Santana et al., 2016; Bohlouli et al., 2019). In plant breeding, therefore, this model may provide considerable biological insights into the mechanisms determining performance in specific environments, making it a worthwhile method for study in future research.

### Complementary “-Omics” Technologies

The rapid advances in “-omics” technologies enable the generation of large-scale “-omics” datasets for many crop species, providing new opportunities to investigate and improve complex traits. The different approaches described in this review offer valuable tools to combine phenomics and genomics data to reveal the underlying genetic basis of longitudinal traits. However, one current challenge is integrating additional “-omics” technologies (e.g., transcriptomics, metagenomics, proteomics, metabolomics, epigenomics) to provide a holistic multi-omics approach to study biological mechanisms and their response to environmental stresses for important agronomic traits.

Recently, methods that combine “-omics” information have been used in some crops to study phenotypic networks for single-point traits, for example, to pinpoint candidate genes and/or loci and predict phenotypic variation (Acharjee et al., 2016; Li et al., 2016; Das et al., 2017; Sheng et al., 2017; Pandey et al., 2018; Jiang et al., 2019). A meta-analysis of the detailed “-omics” datasets regarding longitudinal traits in crops has been limited so far. Baker et al. (2019) characterized the mechanistic connections between the genomic architecture, transcriptomic expression networks, and phenotypic variation of growth curves that underlie the developmental dynamics of plant height in *Brassica rapa*. The combination of multi-omics approaches also seems promising to elucidate senescence processes in model and crop plants (Großkinsky et al., 2018). When joint modeling longitudinal “-omics” data (one or more type of “-omics” data measured over time) the statistical analysis becomes more challenging. Some key points can be found in Sperisen et al. (2015). In general, there is a need to adapt methodologies and experimental designs to explore processes related to the global evolution of biological processes such as growth and development. Despite all these challenges, integrative methods can increase analysis power to find true causal variants, regulatory networks, and pathways. These, in turn, could be incorporated into GS and breeding programs to speed up genetic gains (Suravajhala et al., 2016).

### Deep Learning

Deep learning (DL) is a powerful and highly flexible class of machine learning algorithms based on representation-learning methods that incorporates multiple levels in a non-linear hierarchical learner (Lecun et al., 2015). Essentially, DL is an advanced version of artificial neural networks (ANN) with multiple hidden layers that aims to mimic the human brain functioning (Patterson and Gibson, 2017).

Deep learning has demonstrated its utility in different fields of biological sciences, such as disease diagnosis (Gao et al., 2018), multi-omics data integration (Chaudhary et al., 2018), predicting DNA- and RNA-binding specificity (Trabelsi et al., 2019), and, recently, in plant breeding genomic prediction (Ma et al., 2018; Montesinos-López A. et al., 2018; Montesinos-López O. A. et al., 2018; Montesinos-López et al., 2019). Zou et al. (2019) and Pérez-Enciso and Zingaretti (2019) provide a primer on DL in genomics. The growing interest in DL methods in plant breeding, especially for prediction, may arise from its powerful capability of learning complex non-linear relationships between predictors and responses hidden in big data, usually resulting in higher accuracy when compared with other methods (Montesinos-López A. et al., 2018; Pérez-Enciso and Zingaretti, 2019; Zou et al., 2019). It is important to point out that even though DL can deal with complex scenarios and achieve state-of-the-art accuracy, it requires domain knowledge and large-scale datasets, while the interpretation of the underlying biology is more challenging than for standard statistical models (Zou et al., 2019).

Within the classes of DL, recurrent neural networks (RNN) are designed for sequential or time-series data (Lecun et al., 2015) and may be the most appropriate architecture to model longitudinal traits. An RNN can be thought of as a memory state that retains information on previous data the network has seen and updates its predictions in the light of new information. Thus, besides prediction, RNN has the ability to capture long-term temporal dependencies (Che et al., 2018). Recently, RNNs have achieved astonishing results in many applications with time series or sequential data, particularly in human sciences (Azizi et al., 2018; Che et al., 2018; Lee et al., 2019; Sung et al., 2019; Zhong et al., 2019). Despite its advantages, to our best knowledge RNNs have not been employed in genomic prediction or mapping QTL for longitudinal traits in plant breeding. In the context, versatile DL models for multiple-trait analysis (Montesinos-López O. A. et al., 2018), multiple-environment analysis (Montesinos-López O. A. et al., 2018), and performing simultaneous predictions of mixed phenotypes (binary, ordinal and continuous; Montesinos-López et al., 2019) have been successfully implemented. Such cases are not only encouraging but may lead to future integration of DL and RNNs into the analysis of longitudinal traits in crops. DL is a powerful approach and is likely to transform many domains in plant breeding because it has the potential to handle all the complexities highlighted in this review. Needless to say, further innovation and technology assessment are required to fully enable DL to deal with the unique properties of planting breeding data.

## Author Contributions

FM, KR, and LB conceptualized the manuscript. FM wrote the manuscript. HO, LB, and KR critically revised and improved the manuscript. JV edited section 3 “Modeling Longitudinal Traits.” All authors read and approved the manuscript.

## Conflict of Interest

The authors declare that the research was conducted in the absence of any commercial or financial relationships that could be construed as a potential conflict of interest.

## Footnotes

1 www.cimmyt.org/

## Abbreviations

3D: three-dimensional
3PL: three-parameter logistic
4PL: four-parameter logistic
5PL: five-parameter logistic
ANOVA: analysis of variance
AR-1: first-order autoregressive analysis
BIC: Bayesian information criterion
BLUE: Best Linear Unbiased Estimation
BLUP: Best Linear Unbiased Prediction
CIMMYT: International Maize and Wheat Improvement Center
CT: canopy temperature
CT: computed tomography
DL: deep learning
FA: factor analysis
GWAS: genome-wide association studies
GS: genomic selection
GBLUP: genomic-based BLUP
GNDVI: green normalized difference vegetation index
HTP: high-throughput phenotyping
LiDAR: laser-imaging detection and ranging
MTM: multiple-trait model
MTRRM: multiple-trait random regression model
MANOVA: multivariate analysis of variance
NIR: near-infrared
NDVI: normalized difference vegetation index
PCA: principal component analysis
QTL: quantitative trait loci
RRM: random regression model
RNN: recurrent neural networks
RNDVI: red normalized difference vegetation index
RGB: red-green-blue
ssGBLUP: single-step GBLUP
AIC: Akaike information criterion
UAV: unmanned aerial vehicle.
